# “What makes this a wug?” Relations among children’s question asking, memory, and categorization of objects

**DOI:** 10.3389/fpsyg.2022.892298

**Published:** 2022-08-11

**Authors:** Emma Lazaroff, Haley A. Vlach

**Affiliations:** Department of Educational Psychology, University of Wisconsin-Madison, Madison, WI, United States

**Keywords:** question asking, inquiry, memory, categorization, cognitive development

## Abstract

Children ask many questions, but do not always receive answers to the questions they ask. We were interested in whether the act of generating questions, in the absence of an answer, is related to children’s later thinking. Two experiments examined whether children retain the questions they ask in working memory, and whether the type of questions asked relate to their categorization. Four to ten-year-old children (*N* = 42 in Experiment 1, *N* = 41 in Experiment 2) were shown 12 novel objects, asked three questions about each, and did not receive answers to their questions. Children recalled their questions in the first experiment and categorized variants of the novel objects in the second experiment. We found that children have robust working memory for their questions, indicating that these questions may relate to their subsequent thinking. Additionally, children generalize category boundaries more narrowly or broadly depending on the type of question they ask, indicating that children’s questions may reflect an underlying bias in how they think about the world. These findings suggest that future research should examine questions in the absence of answers to understand how inquiry affects children’s cognitive development.

## Introduction

Children are very inquisitive individuals; research has found that children ask an average of 76 information-seeking questions per hour by the time they reach preschool age (Chouinard, 2007), and the frequency of questions increases as the preschool years progress (Hickling and Wellman, 2001). As a result, researchers have examined whether and how questions help children learn. Findings indicate that children’s questions act as a learning mechanism by allowing them to acquire new information (Kemler Nelson et al., 2004; Frazier et al., 2009). That is, these studies have shown that children change their thinking and/or behavior based upon the answers they receive. The current study takes a different approach to studying children’s questions by examining the cognitive consequences of children’s questions themselves, without the presence of answers. The central goal of the current experiments was to determine if and how the act of generating questions relates to children’s subsequent thinking.

### Questions as a learning mechanism

How do questions help children learn? One proposal is that children’s questions are information-seeking in nature (Kemler Nelson et al., 2004; Chouinard, 2007; Frazier et al., 2009; Ronfard et al., 2018). That is, children learn by having their questions answered. Indeed, children ask questions to gain information that is not immediately available to them. For instance, children spontaneously engage in inquiry when information about objects or entities is not provided by older children or adults (Callanan and Oakes, 1992; Hickling and Wellman, 2001). Moreover, children expect to receive different types of information for different types of items and ask their questions accordingly (Greif et al., 2006). When children receive insufficient information, they follow up with additional questions to receive the information most useful to their learning (Kemler Nelson et al., 2004; Frazier et al., 2009). Children not only seek appropriately informative explanations to their questions, but also prefer and learn from these explanations by recalling this information later (Frazier et al., 2016). Finally, children learn from questions even when they are not the ones asking them; young children’s word learning depends in part on the content of questions asked by others (Luchkina et al., 2020). In brief, children seek information through asking questions; by receiving information, children then use this knowledge to help them learn about the world around them.

Children ask many kinds of questions, such as about visual features, functions or behaviors, locations, people, events, cultural conventions, verbal labels, and causal relations (Tizard et al., 1983; Callanan and Oakes, 1992; Chouinard, 2007). Some questions are purely fact-based, such as “What is the capital of Wisconsin?” However, other questions are not fact-based in nature and may be the basis of an inference that then leads to learning, such as “Why did you move to Wisconsin?.” For instance, in the 20-questions paradigm/game, children guess the answer to a problem by asking questions one at a time that are answered with a “yes” or “no.” As another example, Ruggeri and Lombrozo (2014,^2015^) had children ask questions to determine the answer to problems, such as discovering why a man was late for work. Children were found to adapt their questions over time to make inferences that lead to greater information gain (e.g., asking “Did something happen at home?” instead of “Was he late because he overslept?”). Additionally, children ask more of these broader-scope questions with age (Mosher and Hornsby, 1966). By asking each question, children are making an inference about why the man was late for school, and the answer to this inference-based question guides their future inferences and learning. Thus, children ask a variety of questions for a multitude of reasons: to learn the answers to facts, solve problems, and categorize the world.

The process of generating questions becomes more efficient across development. Preschool and kindergarten-age children often struggle to generate questions spontaneously (Jones et al., 2020). As children reach the early elementary years and find it easier to generate questions, these questions tend to be hypothesis-scanning in nature, resulting in less information gain (Mosher and Hornsby, 1966; Herwig, 1982; Ruggeri and Feufel, 2015; Ruggeri and Lombrozo, 2015). By age 10, children begin reliably generating more informative, constraint-seeking questions (Ruggeri and Lombrozo, 2015; Ruggeri et al., 2016). Several cognitive processes likely play an instrumental role in the development of children’s question asking, such as their categorization abilities, executive function, metacognition, and probabilistic reasoning (Jones et al., 2020). For instance, Legare et al. (2013) found that preschoolers who demonstrated more cognitive flexibility during object categorization also generated more informative questions. Additionally, more informative questions may require more advanced categorization abilities: older children ask more of these questions effective for object categorization (Ruggeri and Feufel, 2015), and supporting children’s categorization performance also supports their ability to ask informative questions (Ruggeri et al., 2021).

There may also be age-related biases in the information that children seek through their questions. For instance, research has long found that preschool-aged children primarily use feature-based information over other types of information when categorizing and generalizing objects (Keil, 1989; Gentner and Rattermann, 1991; Merriman et al., 1993; Fisher et al., 2015; Murphy et al., 2021). Furthermore, research on children’s question-asking indicates that even if children ask questions seeking multiple types of information, children still frequently seek feature-based information through their questions (Greif et al., 2006; Chouinard, 2007; Legare et al., 2013; Ruggeri and Feufel, 2015). While there is a developmental shift in the types of information children use to categorize or seek through their questions—for example, that children categorize based on abstract relational information and ask more constraint-seeking questions with age (Gentner, 1988; Rattermann and Gentner, 1998; Ruggeri and Lombrozo, 2015; Ruggeri et al., 2016)—this prior work suggests that children have underlying biases about the world. These biases may impact children’s higher-order cognition (e.g., their categorization) and the questions they choose to ask.

In studies examining what children learn via inquiry, children are generally provided information and/or a direct answer to their question during cognitive tasks. Consequently, it is hard to determine whether children’s questions themselves are related to their thinking and/or inferences. When asking fact-based questions, children likely need to hear the answer to learn something new. For instance, someone would need to tell children that Madison is the capitol of Wisconsin to learn anything from the question “What is the capital of Wisconsin?” However, what about other types of questions? It could be that the act of question asking itself is related to this change and the process of inference making. Another possibility is that questions themselves may not matter at all; simply being provided the information, without having to ask a question, could have yielded the same behavioral response. According to existing theoretical frameworks of epistemic questions as information-seeking behaviors (Chouinard, 2007; Ronfard et al., 2018), children who do not receive answers will ask another question, explore themselves to find the answer, or stop asking questions altogether. That is, the issue of whether questions themselves are even correlated with cognition, or if receiving information alone is sufficient, is never addressed.

Are there cognitive consequences to children’s question asking in the absence of answers? There is little research to date on the act of generating questions. Most of what we know about children’s question asking comes from observation-based research (Sully, 1896; Piaget, 1926; Chouinard, 2007; Ronfard et al., 2018). In early logs of young children’s everyday behavior, Piaget (1926) and Sully (1896) viewed children’s questions as informing how they might see or think about the world as they are asking such questions. However, even the questions asked in these logs were asked in the presence of answers. More recent logs have gone beyond answered questions and revealed that children only receive answers to their questions approximately 71% of the time in naturalistic settings (Chouinard, 2007). Thus, to fully understand how inquiry shapes children’s cognitive development, we also need to begin focusing on the act of generating questions itself. First, however, we need to examine whether question asking and children’s cognition are correlated before we can isolate any potential direct cognitive consequences of question asking. In sum, the central goal of this work was to determine whether the act of generating questions is related to children’s cognition. This work addressed a gap in the literature on children’s inquiry by serving as a first step in isolating the specific mechanisms by which questions themselves may act as a learning mechanism.

### Current study

In the current study, we examined whether and how children’s question asking is related to cognition by testing their thinking after a question-asking period. Given that there are a plethora of ways in which questions could impact cognition (Kemler Nelson et al., 2004; Chouinard, 2007; Frazier et al., 2009), we started this process by looking at two key cognitive capacities: working memory and categorization. In Experiment 1, we examined whether children can retain the questions they generate in working memory. We hypothesized that short-term memories could serve as the foundation for how questions relate to later cognitive development. Children may draw upon their memory for the questions they asked to make inferences, such as during problem solving. For instance, if a child asks the question “Why does this wug have orange feet?,” they may later try to determine if other wugs also have orange feet due to their memory of asking a question about features. Alternatively, children’s questions may be rapidly forgotten and thus not impact children’s memory or cognition. In other words, children may view the questions they ask as irrelevant for their learning and forget accordingly. Indeed, children rapidly forget irrelevant information to promote their learning of new information (Vlach, 2014). In sum, if children retain their questions in their working memory, this suggests that questions themselves are related to children’s cognition. Conversely, if children’s questions are rapidly forgotten, question asking is likely to be unrelated to children’s future thinking and/or behavior.

To foreshadow the results of Experiment 1, we observed that children had strong working memory for their questions, which suggests that children may use these memories to make inferences during higher-order cognitive processes. Thus, the second way we studied the role of question asking in children’s learning was through examining whether question asking may also have cognitive consequences for higher-order cognition. Specifically, in Experiment 2, we looked at children’s categorization of the items they asked questions about. We chose to look at the relation between children’s questions and categorization because categorization represents how children make inferences about the world (Kalish et al., 2015; Vlach, 2016), and previous research has suggested that categorization abilities are closely tied to children’s question asking (Legare et al., 2013; Ruggeri and Feufel, 2015; Ruggeri et al., 2021). In particular, in Experiment 2 we examined whether there were relations between the content of children’s questions and subsequent generalization of category boundaries.

We hypothesized that asking certain questions may relate to how children generalize. For instance, children may ask feature-based questions because they may later tend to generalize based on features. If this is indeed the case, we expected to see a relation between the number of feature-based questions children ask and how broadly or narrowly they generalize category boundaries based on these features. Moreover, we expected to observe a relation between category-based questions (e.g., “Is this wug a type of bird?”) and children’s generalization of category boundaries. Specifically, we predicted that feature-based questions would be related to narrower categorization, and that category-based questions would be related to broader categorization behavior. We tested this hypothesis by looking at how broadly or narrowly children categorized based on the numbers of differing features on novel objects. If we did not find these results, it would provide support for alternative hypotheses. For instance, the nature of the questions that children ask may have no relation to how they generalize category boundaries. There may also be an external, unmeasured factor that impacts both the questions children choose to ask and their later categorization. Taken together, these two experiments provided a first step of examining whether the act of generating questions relates to children’s subsequent learning.

## Experiment 1: Children’s working memory for questions asked

We first examined whether question asking would be related to children’s working memory. That is, in the first experiment we asked: Do children retain the questions they ask in their working memory? We chose to start with this lower-level process because working memory for questions could serve as the foundation for how the act of asking questions affects higher-order cognitive processes, such as categorization or problem solving.

### Materials and methods

#### Participants

The participants were 42 4–10 year-old children (17 females, *M* = 6 years 11 months, median = 6 years 8 months, range = 4 years 0 months to 10 years 5 months). This age range was chosen because it spans the time in which children begin successfully generating questions to the time when they become adept at using efficient question-asking strategies (Jones et al., 2020). We included a broad age range to afford exploratory analyses of whether there are developmental changes or underlying biases in the ways in which children’s question asking relates to their cognition. Indeed, previous studies on children’s question asking also included broad age ranges to investigate developmental changes (e.g., Denney, 1972, 1975; Courage, 1989; Ruggeri and Lombrozo, 2015; Ruggeri et al., 2019).

Effect sizes were gathered from studies on children’s question asking with this age group, which had consistently large effect sizes (*ƞ^2^s* ≥ 0.14; e.g., Greif et al., 2006; Chouinard, 2007; Mills et al., 2010, 2012). Using a medium effect size of *d* = 0.5, a power analysis for a two-tailed t test with *α* = 0.05 revealed that we needed at least 34 participants to have 80% power to observe an effect. 92.86% of parents (*N* = 39) provided demographic data about their child and family. Further demographic information is provided in Appendix A; children came from predominantly white middle- to upper-SES families. Children were recruited from local preschools and elementary schools or came to the lab to participate in the study. Children received a storybook as a thank you for their participation in the study. An additional fourteen children were excluded from analysis due to inability to follow directions^1^ (i.e., did not ask any questions for the duration of the experiment, *N* = 10, and inability to pay attention to the task, *N* = 4).

#### Apparatus and stimuli

**Figure 1:**
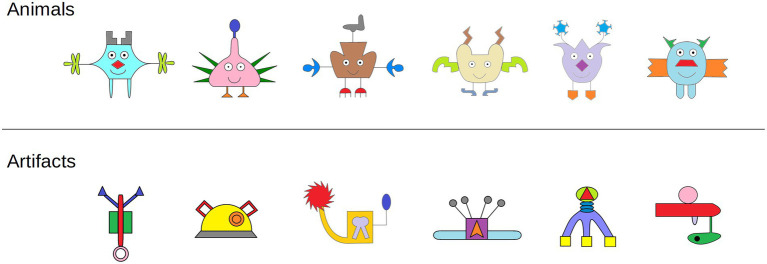
All stimuli from Experiments 1 and 2.

The experiment was administered on an iPad. Visual stimuli consisted of 12 digital drawings of novel objects created using InkScape (Version 0.92.3; Hurst, 2018) presented individually on the iPad screen. The stimuli are shown in Figure 1. Six drawings were artifacts and six drawings were animals. The presentation order of the novel objects was counterbalanced across four random orders; children were randomly assigned to one of these four orders. Auditory stimuli consisted of pseudo-words (e.g., “wug”) presented aloud to the children by the experimenter to eliminate the possibility of known words influencing children’s questions about the items. All novel words were consistent with the phonotactic probabilities of American English. Each visual stimulus was paired with a pseudo-word, and these pairings remained consistent across all conditions and participants. Participants’ responses were recorded using an audio recording device.

#### Design

**Figure 2:**
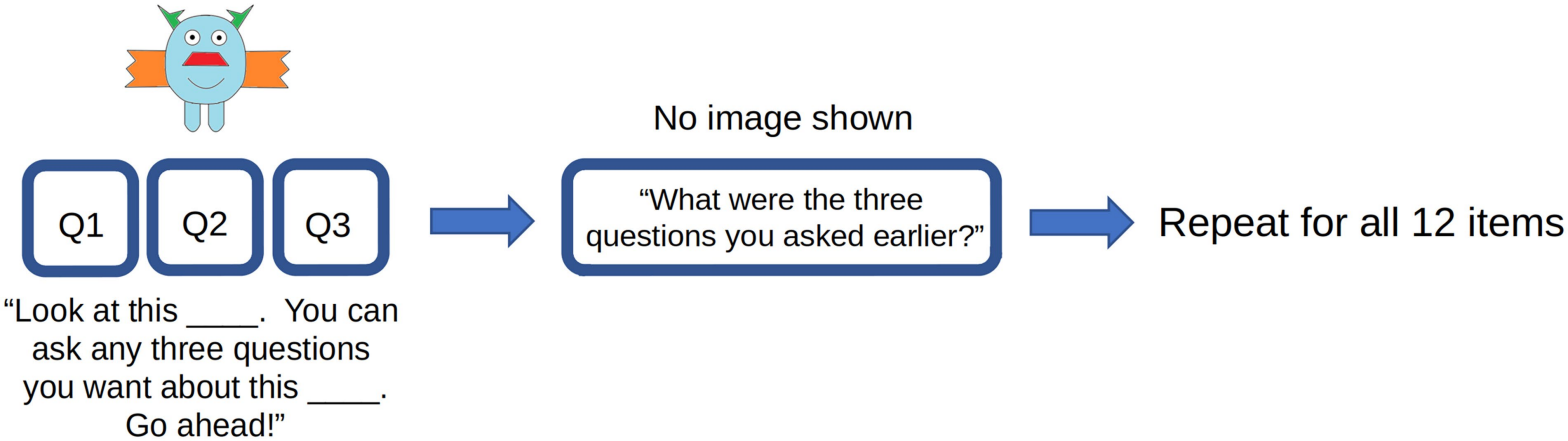
Examples of stimuli and procedure for Experiment 1. Participants asked three questions about a novel object, immediately recalled those questions, then repeated this procedure for all 12 novel objects.

All children completed trials that involved both asking questions about the novel objects and recalling the questions they just asked. Thus, the study was a within-subjects design in which all participants viewed the same 12 novel objects, asked questions about these objects, then recalled these questions (Figure 2).

#### Procedure

All experiments were first approved by the University of Wisconsin-Madison Education and Social/Behavioral Sciences Institutional Review Board; the project title was “The Development of Children’s Memory for Objects and Words,” project ID: 2015–0826. The stimuli and procedure are outlined in Figure 2. Children were presented with one novel object at a time and were instructed to ask any three questions about each item that they wished. For instance, the experimenter would say: “Look at this wug. You can ask any three questions you want about this wug. Go ahead!” Children did not receive answers to their questions. All children asked exactly three questions for each item: the experimenter kept track of the number of questions children asked and prompted children to ask a third question if they had only asked one or two. After asking the three questions for each item, children were prompted to recall their questions immediately by being asked, “What were the three questions you just asked?” Children saw the novel object in question on the screen while recalling their questions. The experimenter then advanced to the next item, in which children were again prompted to ask and then recall their questions. The process of question asking took an average of approximately 10 s per item, with 10 s passing between children asking the last question and recalling the questions they asked. During the 10-s gap between question 3 and recall, children were informed they had asked three questions and were also informed that they were going to do something else. Children completed this procedure for all 12 novel objects.

#### Coding and analysis of the questions

We developed a coding scheme to determine exactly what types of questions children asked. The types of questions children asked were coded as follows: a question was coded as a feature-based question if it contained elements referring to the novel object’s physical appearance (e.g., the words “green thing on the top” in the question “What is that green thing on the top?”). A question was coded as a category-based question if it contained elements referring to the novel object’s category membership (e.g., the words “type of bird” in the question “Is this wug a type of bird?”). Questions could also be coded as being both feature-based and category-based (e.g., “Do all wugs have orange feet?”; the words “all wugs” refers to category membership, and the words “orange feet” refer to features). We also coded for the following question types: function, behavior, ambiguous, location, creation, and social relevance-based questions. However, we did not explore these in Experiment 1 or 2 because they were sparsely asked. Inter-rater reliability across all question categories, including superordinate and subordinate questions was 90%. Examples of all question types are listed in Appendix B.

We also coded category-based questions as superordinate and subordinate. Superordinate category-based questions were defined as questions that related solely to category membership (e.g., “Is this wug a type of bird?”). Subordinate category-based questions were defined as questions that explicitly referenced specific feature-based information. For instance, the question “Do all wugs have orange feet?” was coded as subordinate because the feature of “orange feet” was explicitly mentioned. Alternatively, this same question “Do all wugs have orange feet?” could potentially also be interpreted as a superordinate question, because it refers to the broader category of “all wugs” in addition to explicit feature information. However, for the purpose of this study, this question would still be coded as subordinate because it explicitly mentions the feature “orange feet.” We coded children’s questions in this way because it aligns with how feature and category-based information was coded in previous research on children’s question-asking behavior (e.g., Greif et al., 2006; Chouinard, 2007).

Additionally, questions were coded as “correct” if they were recalled mostly word-for-word. Children did not always recall questions completely word-for-word, but nearly always recalled them mostly word-for-word. For instance, if a child asked, “Why does it have purple feet?” but recalled their question as “Why does it have those purple things down there?” while referring to the feet, the question recall was coded as correct. As with the question types, inter-rater reliability was 90% when we coded for correct versus incorrect question recall.

### Results

We were interested in whether children retained the questions they asked in working memory. We hypothesized that children would remember the majority of their questions, as these short-term memories could serve as a foundation for later thinking and learning. To test this hypothesis, we calculated the mean percentage of questions children remembered out of all of the questions asked. We found that children indeed have robust working memory for their own questions: on average, children were able to remember 77.5% (*SD* = 18.3) of the total number of questions they asked. No child remembered less than one-third of the questions they asked (range = 12–36). These results suggest that children attend to and retain the questions that they ask in working memory, possibly serving as foundational representations for later thinking and learning.

**Figure 3:**
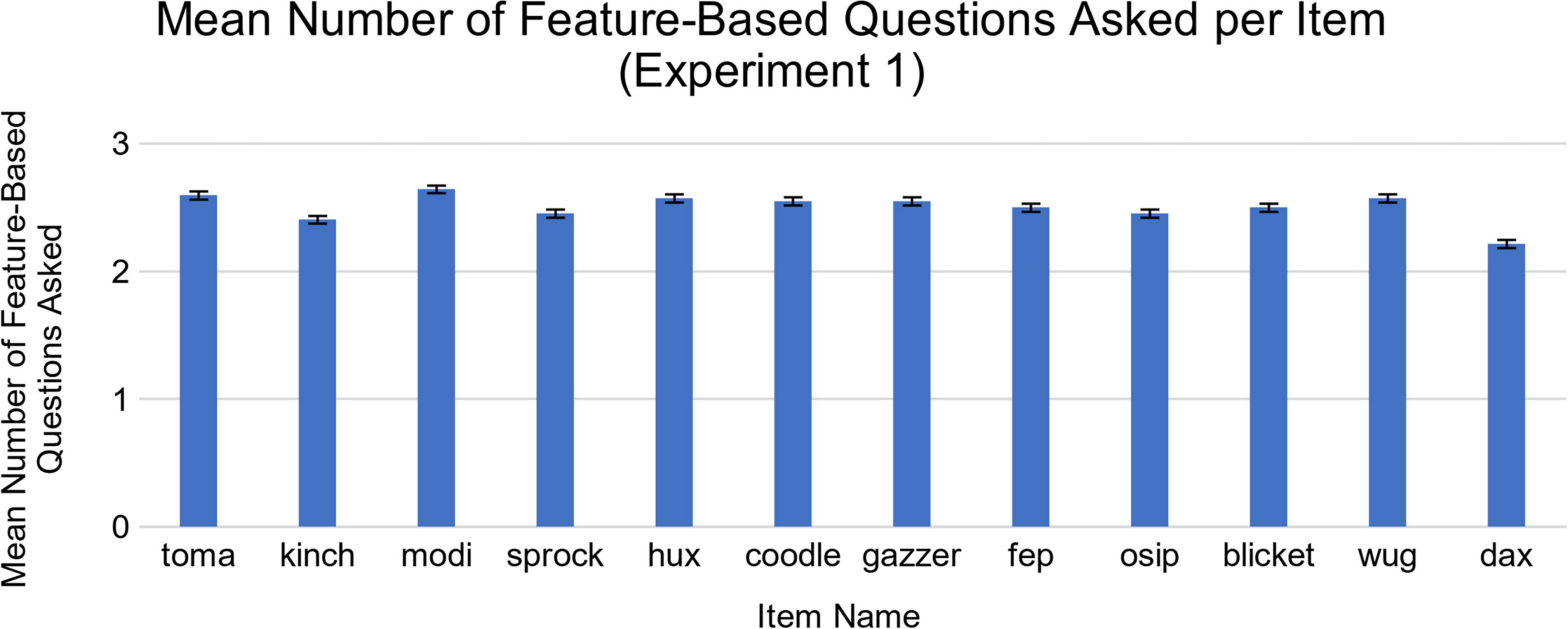
Mean number of feature-based questions asked for each novel object in Experiment 1. There were no significant differences across the items.

**Figure 4:**
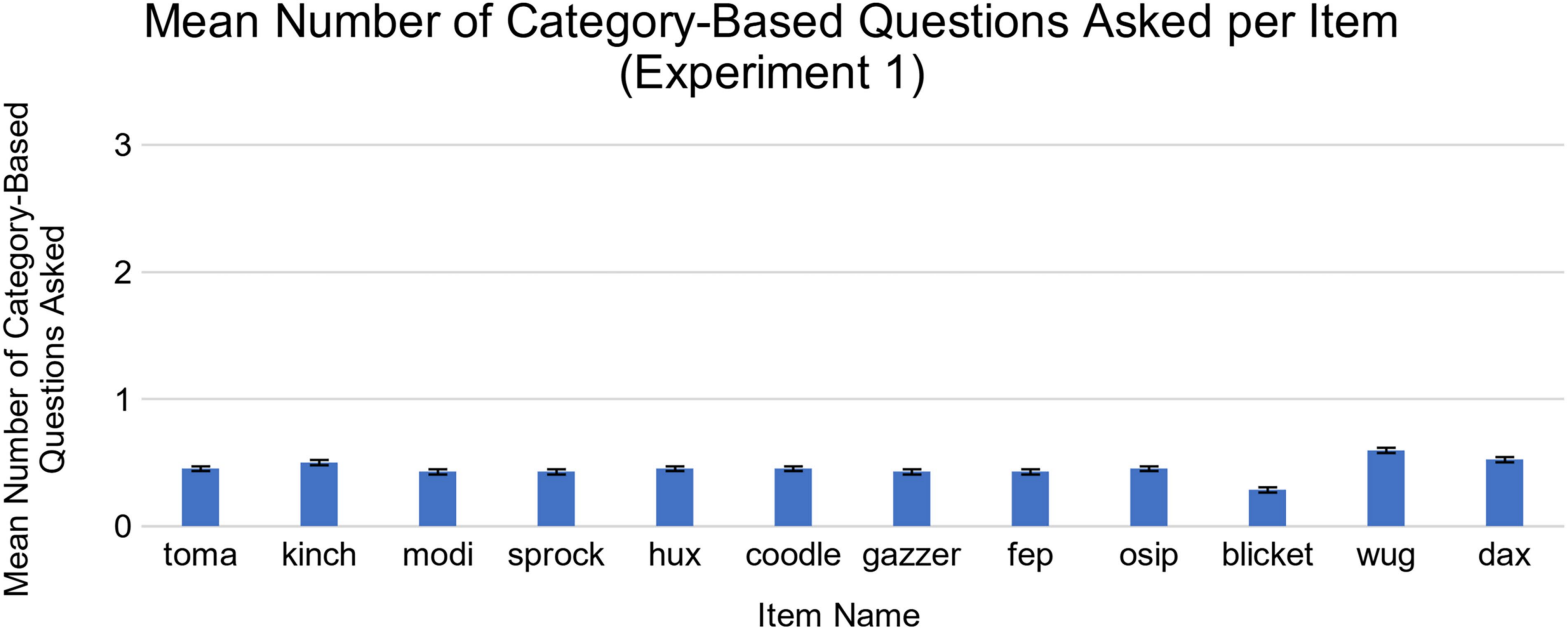
Mean number of category-based questions asked for each novel object in Experiment 1. There were no significant differences across the items.

We then examined whether there were differences in children’s memory for the different types of questions they asked. First, we conducted an exploratory analysis of the nature of children’s questions by examining the different types of questions children asked. We found that the majority of the questions children asked were about features, such as “What is that green thing on the top?” [*M* = 29.98 (out of 36), *SD* = 8.22, range = 5–36]. Children also asked other types of questions, such as category-based questions; for example, “Is this wug a type of bird?” (*M* = 5.52, *SD* = 7.64, range = 0–33). On average, children asked more feature-based questions than category-based questions, *t*(41) = 12.02, *p* < 0.001, *d* = 1.85, 95% CI [20.34, 28.56]. All 42 children asked feature-based questions, while 25 children asked category-based questions. Thus, we chose to focus on differences in memory for feature and category-based questions for Experiment 1. No single item elicited significantly more feature or category-based questions than the others (Figures 3, 4). Further details about how questions were coded are included in the Results section of Experiment 2.

Next, we looked separately at the percentages of category-based and feature-based questions that children remembered. Results revealed that children remembered a similar proportion of category-based questions (*M* = 87.08%, *SD* = 15.39) and feature-based questions (*M* = 84.12%, *SD* = 14.58), *t*(25) = 0.806, *p* = 0.428, *d* = 0.16, 95% CI [−4.6, 10.53]. One might expect children to have better memory for feature-based questions, as children viewed the novel objects when they were asked to recall their questions. However, because children remembered a similarly high proportion of category-based questions, this suggests that category-based questions may relate to children’s thinking and behavior. This is discussed in more detail in the General Discussion.

Looking further at the types of questions children asked, we examined the stability of children’s question-asking; that is, whether children asked more or less of a specific type of question over the course of the experiment. About 63% of children changed (increased or decreased) the number of feature-based questions they asked between the first and second half of the experiment, with the remaining 37% of children asking the same amount of feature-based questions in each half. Similarly, 61% of children changed the number of category-based questions they asked, with the remaining 39% asking the same amount of category-based questions in each half of the experiment (Appendix C). Children who adapted their question-asking behavior did so only slightly, asking 2–3 more questions of the same type and 1–2 fewer questions of the same type over time (Appendix D). In brief, children’s question-asking behavior remained relatively stable throughout the experiment, with most children only slightly adapting their question-asking behavior over time.

We also separated children’s inquiry behavior based on age, separating them by preschool age (under age 6) and elementary school age (ages 6 and up) because we wanted to determine whether there were developmental differences between these age groups in the types of questions asked. We split children into these age groups based on the age of transition to formal schooling, as children who have entered formal schooling are more likely to have received explicit instruction on question-asking. Indeed, the Next Generation Science Standards and the Common Core State Standards in ELA and Mathematics all include the act of asking questions as an important skill that K-12 students should know and do (National Governors Association Center for Best Practices and Council of Chief State School Officers, 2010; National Research Council, 2012). Older children in this age range also generally have more experience in question asking and ask different types of questions than younger children (Mills et al., 2010, 2011; Legare et al., 2013; Ruggeri and Lombrozo, 2015; Ruggeri et al., 2016).

Analyses splitting children into these age groups found that the average number of category-based questions asked differed significantly based on age: specifically, older children in the sample asked more category-based questions (*M* = 7.57, *SD* = 8.5) than younger children (*M* = 1.43, *SD* = 2.62), *t*(40) = 2.63, *p* = 0.012, *d* = 0.86, 95% CI [1.42, 10.87]. Older children also asked fewer feature-based questions (*M* = 27.54, *SD* = 9.14) compared to younger children (*M* = 34.86, *SD* = 1.1), *t*(40) = −2.97, *p* = 0.005, *d* = −0.97, 95% CI [−12.3, −2.34]. We also conducted these analyses with age in months as a continuous variable, which revealed similar results: there was a positive correlation between age and the total number of category-based questions asked (*r* = 0.62, *p* < 0.001) and a negative correlation between age and the total number of feature-based questions asked (*r* = −0.38, *p* = 0.012). Therefore, it appears that there are developmental differences in the types of questions children choose to ask. These findings are further explained in the Discussion.

We also examined whether order or item effects were present in children’s working memory for questions. No item effects were found; that is, children’s memory for their questions was similar across all 12 novel objects in the experiment. When examining order effects, paired-samples t-tests revealed that children were more likely to remember the third question they asked compared to the second or first question for a given item (see Appendix E). Additionally, children remembered more questions from the first half of the experiment (*M* = 14.68, *SD* = 2.95) than the second half (*M* = 13.2, *SD* = 4.19), *t*(40) = 3.34, *p* = 0.002, *d* = 0.52, 95% CI [0.19, 0.85].

Finally, we examined whether children’s knowledge of an upcoming memory test affected their working memory. Children may have retained questions in working memory only because they knew that they would be tested on them. To test for this possibility, we compared performance on the first item, when children did not know that they would be tested, to performance on the last item, when children had likely learned that they would be tested. There was no significant difference in children’s memory for their questions for the first item (*M =* 2.5, *SD = 0*.8) compared to the last item (*M =* 2.21, *SD =* 1) in the experiment, *t*(41) *=* 1.58, *p = 0*.12, *d* = 0.24, 95% CI [−0.07, 0.55]. Thus, children also remember their questions even when they do not know that they will be prompted to recall them.

In brief, children indeed have strong working memory for their questions. This suggests that children’s questions are prioritized in lower-order cognitive processes. This also raises the question of whether children’s question asking is also related to higher-order cognitive processes, such as categorization. If the act of asking questions relates to children’s lower-order cognition, the next step is to determine whether there is a relation between children’s questions and how they think about the world. As mentioned earlier, our exploratory analysis from Experiment 1 found that children asked mostly feature-based or category-based questions. Thus, we also focused on these types of questions in Experiment 2. Experiment 2 examined whether there are relations between the types of questions asked (i.e., feature- vs. category-based) and how children categorize novel objects.

## Experiment 2: Children’s categorization after the questions they ask

Experiment 1 showed that children remember the questions they ask. These findings raise the question of whether children’s question asking may also relate to their higher-order cognition. One possibility is that children could draw on memories for their questions to make inferences during higher-order cognitive processes such as categorization. Experiment 2 tested this possibility by examining whether the type of questions children ask related to the manner in which they categorize objects. We hypothesized that children would generalize category boundaries more narrowly or more broadly depending on whether they asked more feature or category-based questions. If children categorize differently based on the types of questions they ask, these results would suggest that question asking may be correlated with how children conceptualize the world.

### Materials and methods

#### Participants

The participants were 41 4–10 year-old children (21 females, *M* = 6 years 9 months, median = 6 years 10 months, range = 4 years 5 months to 10 years 2 months). 95.12% of parents (*N* = 39) provided demographic data about their child and family. Further demographic information is provided in Appendix A; children came from predominantly white middle- to upper-SES families. Children were recruited from local preschools and elementary schools or came into the lab to participate. Children received a storybook as a thank you for their participation in the study. Twelve children were excluded from analysis due to inability to follow directions (i.e., did not ask any questions for the duration of the experiment, *N* = 5, inability to pay attention to the task, *N* = 5, and failure to finish all trials of the study, *N* = 2).

#### Apparatus and stimuli

The apparatus and stimuli were identical to that of Experiment 1.

#### Design

All children completed trials that involved both asking questions about the novel objects and categorizing these objects. Thus, the study was a within-subjects design in which all participants viewed the same 12 novel objects, asked questions about these objects, then categorized these objects.

#### Procedure

**Figure 5:**
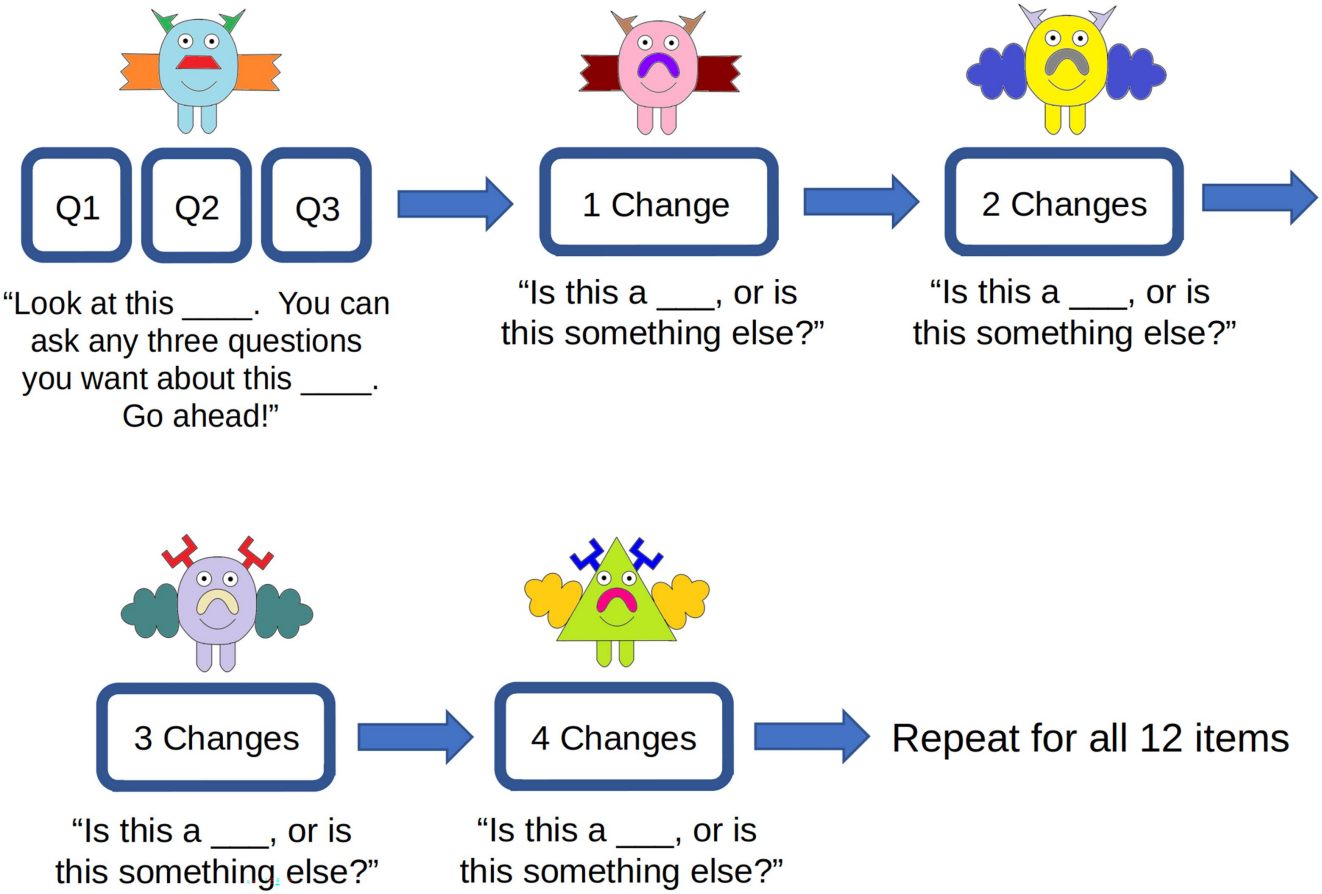
Examples of stimuli and procedure for Experiment 2. Participants asked three questions about a novel object, completed four categorization trials for that novel object, then repeated this procedure for all 12 novel objects.

All experiments were approved by the University of Wisconsin-Madison Education and Social/Behavioral Sciences Institutional Review Board; the project title was “The Development of Children’s Memory for Objects and Words,” project ID: 2015–0826. The stimuli and procedure are outlined in Figure 5. Children were presented with the same 12 novel animals and artifacts as Experiment 1 and underwent the same question-asking procedure as Experiment 1. Immediately after asking the questions about each novel object, children were presented with four different versions of the item they just saw, one at a time. The dissimilarity across each item was gradually increased: the first item had one featural change (e.g., a different shaped body part), the second item had two featural changes, and the number of featural changes gradually increased until the fourth item with four featural changes (Figure 5). All novel objects had exactly four changeable features. All features were different from one another and oriented in different positions, so there was no identical order in which features changed across items. When presented with each item, children were asked, “Is this a wug or is this somet0hing else?.” The purpose of this was to determine the threshold or boundary at which children identify the novel object as belonging to a different category than the initial item.

#### Coding and analysis of the questions

We used the same coding scheme for children’s questions in Experiment 2 as we did in Experiment 1.

### Results

We were interested in whether the types of questions children asked was related to their generalization of category boundaries. We hypothesized that children would generalize category boundaries more broadly or more narrowly depending on the type of questions they asked. Thus, the first step was to determine the nature of children’s questions so that they could be compared to children’s generalization of category boundaries. We therefore started our analysis by calculating the descriptive statistics for question types.

**Figure 6:**
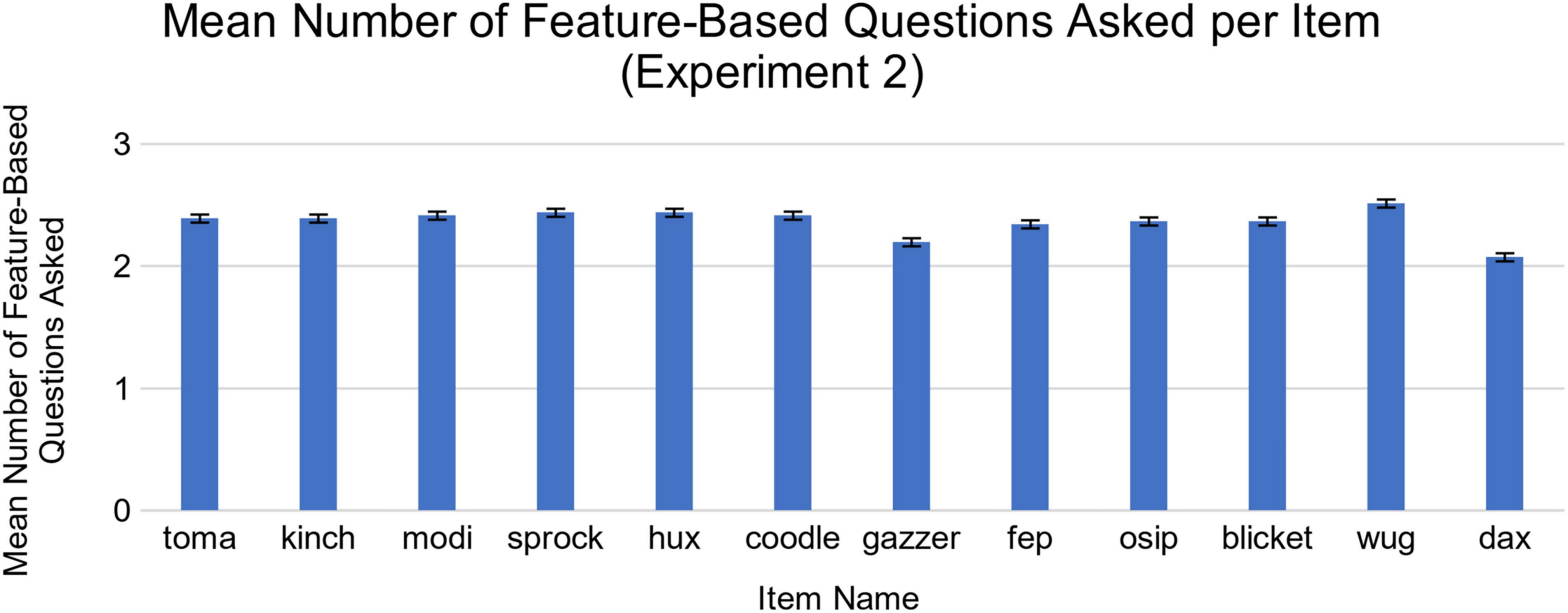
Mean number of feature-based questions asked for each novel object in Experiment 2. There were no significant differences across items.

**Figure 7:**
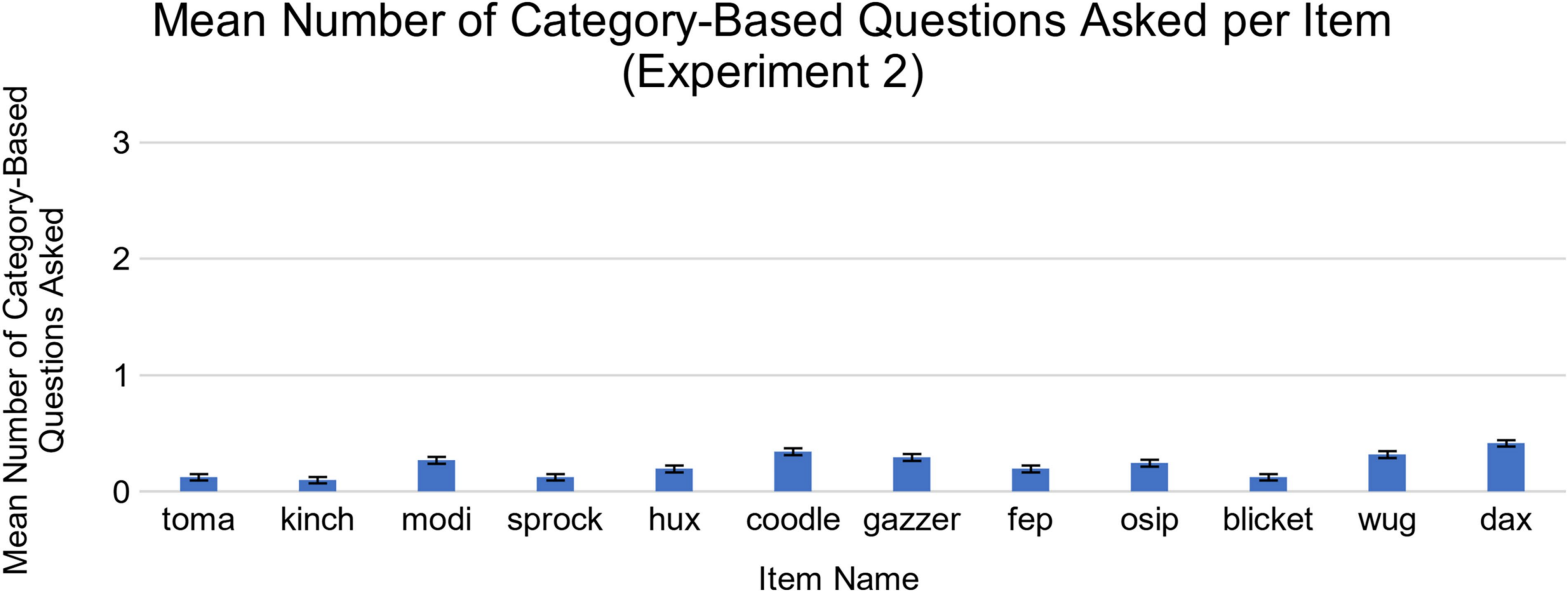
Mean number of category-based questions asked for each novel object in Experiment 2. There were no significant differences across items.

Similar to the exploratory analysis from Experiment 1, we found that children’s questions were mostly about features, and that children asked feature-based questions far more often than category-based questions on average. Out of 36 questions asked during the course of Experiment 2, children asked an average of 28.34 feature-based questions (*SD* = 10.83, range = 0–36) and 2.76 category-based questions (*SD* = 4.1, range = 0–13), *t*(40) = 13.34, *p* < 0.001, *d* = 2.08, 95% CI [21.71, 29.46]. No single item elicited significantly more feature or category-based questions than the others (Figures 6, 7). While children did ask more feature-based questions on average, the number of children who asked solely feature-based questions (*N* = 21) and the number who included category-based questions (*N* = 19) did not differ significantly. As with Experiment 1, we found that most children adapted their question-asking behavior slightly over time (See Appendices F, G).

We also conducted age-related analyses as with Experiment 1. We found that the average number of category-based questions asked differed significantly based on age group: specifically, older children (ages 6 and up) asked more category-based questions (*M* = 3.74, *SD* = 4.46) than younger children (under age 6; *M* = 0.27, *SD* = 0.91), *t*(39) = 2.95, *p* = 0.005, *d* = 1.15, 95% CI [0.29, 1.68]. This age difference was not found for feature-based questions, *t*(39) = −1.5, *p* = 0.142, *d* = 0.52, 95% CI [−1.17, 0.17]. When we conducted these analyses with age in months as a continuous variable, there was a positive correlation between age and category-based questions asked and a negative correlation between age and feature-based questions asked, but neither were significant. In sum, we saw similar developmental patterns as Experiment 1, with children asking mostly feature-based questions and asking more category-based questions with age.

Finally, we tested for order effects as with Experiment 1 by determining if there were differences between when children asked certain types of questions (e.g., first vs. third question) and how they categorized. We conducted hierarchical linear regressions with mean last category endorsement as an outcome variable and number of feature and category-based questions for the first and third questions as predictors, and found that the nature of the third question was no more predictive of categorization behavior than the nature of the first question, *p*s > 0.05. In brief, order effects analyses from both Experiments suggest that recency or practice effects did not contribute to the overall pattern of results.

#### Categorization task

Next, we examined the descriptive statistics for the categorization task. It was important to determine that there were clear differences between each variant of the target novel object to ensure that there was diversity in children’s generalization of category boundaries. To investigate whether these differences existed, we conducted a one-way repeated measures ANOVA to determine whether children endorsed objects (i.e., stated that the object was a “wug” rather than “something else”) that looked more like the target (i.e., had less featural changes) over objects that did not. We found a significant effect of object similarity to the target, Wilks’ Lambda = 0.392, *F*(3,33) = 17.08, *p* < 0.001, *η^2^* = 0.61. Children endorsed the object that looked most like the target (one featural change) the most often, followed by the object with two, three, then four featural changes. There was significant variability in which item children endorsed last (See Appendix H). Pairwise comparisons indicated that nearly all pairwise differences were significant (Appendix I). Additionally, there was no correlation between age and children’s mean last category endorsement, *r* = 0.164, *p* = 0.307. We also examined individual items (e.g., did some objects elicit more narrow/broad generalization than others?) and did not observe any item effects. Therefore, there were indeed differences in how children endorse each variant of the target, indicating that the categorization task is a useful measure for determining differences in generalization of category boundaries.

#### Questions in relation to categorization task

**Figure 8:**
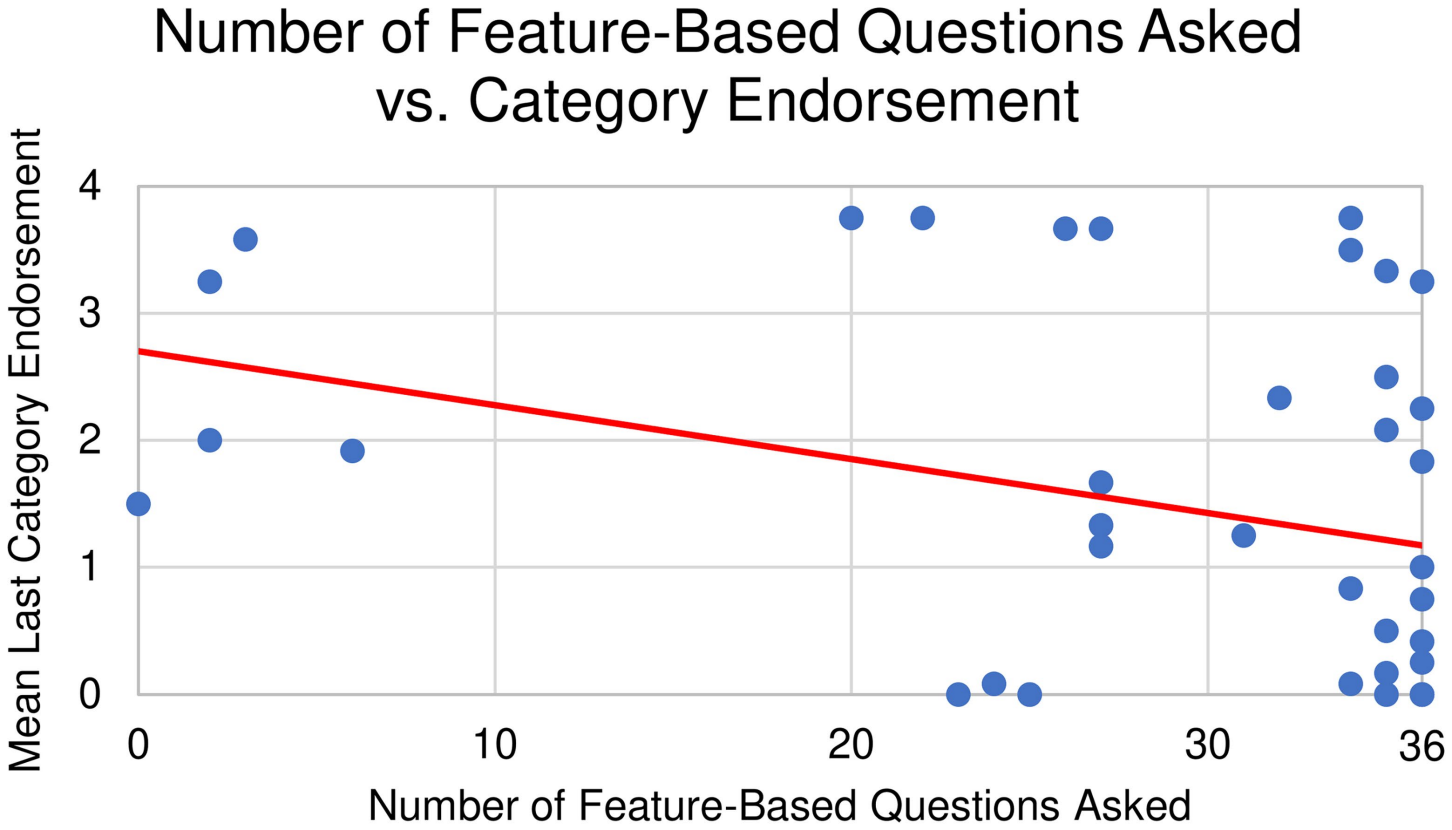
Bivariate correlation between feature-based questions and category endorsement.

**Figure 9:**
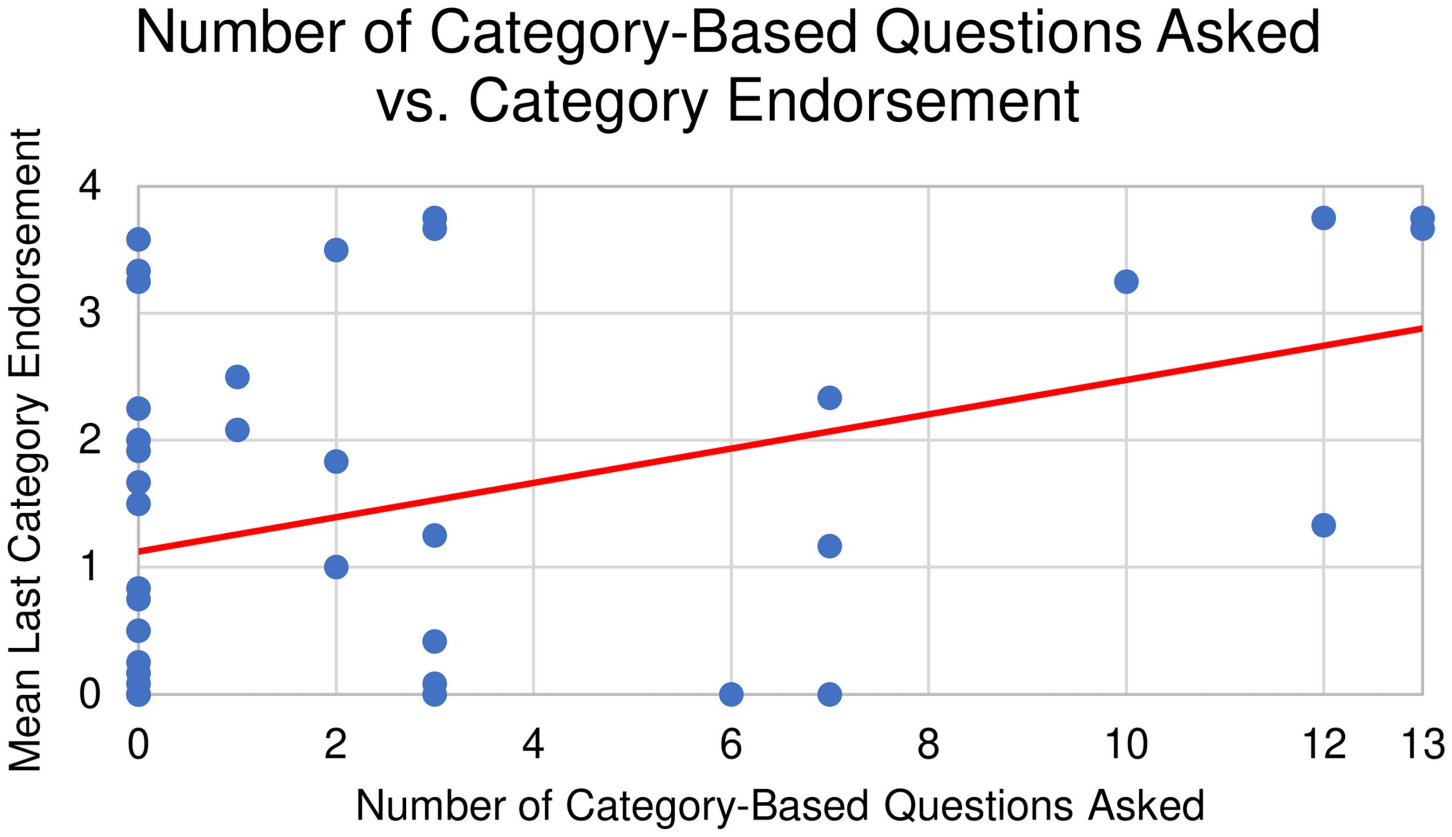
Bivariate correlation between category-based questions and category endorsement.

To test our hypothesis that question type is related to generalization of category boundaries, we conducted bivariate Pearson’s correlations between the total number of feature-based or category-based questions asked and the mean last item endorsed during categorization. We found that when children asked more feature-based questions (i.e., “Why does it have this green part on it?”), they categorized more narrowly. That is, they were less likely to endorse items as belonging to the same category as the initial object (*r* = −0.327, *p* = 0.037; Figure 8). However, when children asked more category-based questions (i.e., “Is this a type of animal?”), they categorized more broadly (*r* = 0.417, *p* = 0.01; Figure 9).

Digging deeper into this analysis, we also conducted independent samples t-tests to determine whether children generalized category boundaries more narrowly or broadly depending on whether they asked superordinate category-based questions, subordinate category-based questions, or both types of questions (see the “Coding and Analysis of the Questions” section in Experiment 1 for information on how superordinate and subordinate questions were coded). Independent samples t-tests revealed no significant differences in categorization behavior based on whether children asked superordinate or subordinate category-based questions. Therefore, these results suggest that when looking at feature-based and category-based questions more generally, question type is related to higher-order cognition, such as how children endorse category membership.

Finally, we fit a mixed-effects logistic regression model predicting children’s likelihood of endorsing a given item as a member of the initial category with age (in months) as a fixed factor, and the number of feature-based questions asked, the number of category-based questions asked, and the number of feature changes from the target item as predictors (Appendix J). To control for repeated measures, we included by-participant and by-item random intercepts. We found that the number of feature-based questions asked, the number of category-based questions asked, and the number of feature changes from the target item all uniquely contributed to children’s category endorsements. Age did not significantly contribute to category endorsement. Specifically, these regression models revealed the same pattern as our correlations: the more feature-based questions children asked or the more feature changes there were from the target item, the less likely they were to endorse an item as a member of the initial category. Children who asked more category-based questions were more likely to endorse an item as a member of the initial category. This suggests that when accounting for subject and item-level effects, feature and category question types and the number of changed features all uniquely predict children’s likelihood of endorsing each item as belonging to the initial category.

## General discussion

The central goal of the experiments was to determine whether the act of generating questions relates to children’s subsequent thinking and learning. Specifically, the studies were designed to determine whether (a) children retain the questions they ask in working memory, and (b) whether the act of asking questions is related to how children think about the world (using generalization of novel object categories). The results revealed that children have a robust working memory for the questions they ask (Experiment 1), and that asking certain types of questions (i.e., feature- and category-based questions) predicts how near or far they generalize category boundaries (Experiment 2). To our knowledge, this study is the first to investigate whether the act of generating questions, in the absence of an answer, relates to children’s cognition. Thus, the primary theoretical contribution of this work is the knowledge that the act of asking questions is correlated with children’s subsequent thinking and behavior.

In Experiment 1, we took the critical first step of examining whether children remember the questions they ask, as these short-term memories could serve as a lower-level foundation for higher-order thinking like categorization. We found that children had strong working memory for these questions—why? There are several possible explanations. First, children may purposefully be trying to attend to and remember the questions they ask so they can ask similar questions again later, with the hopes of eventually getting an answer. Second, children may retain most of their questions in working memory because these questions reflect how they are thinking about the world. That is, children may have good working memory for their questions because these questions are tightly tied to their biases and prior knowledge. For instance, children asked mostly feature-based questions. The fact that they asked mostly feature-based questions could mean that children at this age are biased to think about the world in terms of features, and thus retain feature-based questions in working memory. Indeed, children have stronger memory for information that is in line with their prior knowledge or biases (e.g., Chi and Ceci, 1987; Vlach, 2016).

Despite this bias, however, children in Experiment 1 remembered an equally high proportion of category-based questions. This further supports the idea that these questions reflect how they think about the world: since children viewed the item again when recalling the questions, they may have recalled more feature-based questions. Instead, they also recalled most questions containing category-based information that was not apparent when looking at the item. Category-based questions may also require more cognitive effort to generate and remember since they are not in line with children’s bias to focus on features. This may explain their strong memory for these questions: previous research indicates that tasks requiring more cognitive effort may support memory for information (Tyler et al., 1979; Bjork, 1994; Pyc and Rawson, 2009). However, the current research did not examine children’s long-term memory for these questions. If children indeed use their questions to guide their learning, then they should not only retain these questions in working memory as shown in Experiment 1, but they should also encode and retain these questions in long-term memory. Thus, future research should investigate whether children have memory for their questions over longer timescales.

In addition, we also found that children’s working memory for observations was similar to their memory for questions in Experiment 1. In other words, the children who failed to generate questions and instead made observations also remembered the majority of their observations. This finding suggests that children’s questions may be one of multiple factors that relate to both lower-order working memory and higher-order thinking such as categorization. Children’s observations may be an equally important factor and may also reflect children’s prior knowledge or biases. In short, children who cannot spontaneously generate questions when prompted may rely on their working memory for other sources, such as their self-generated observations, to make inferences during higher-order cognitive processes.

Experiment 2 tested the possibility that the act of asking questions relates to inferences made during categorization. We found that children who asked more feature-based questions generalized more narrowly whereas children who asked more category-based questions generalized more broadly. Why did we find that children’s questions related to their categorization behavior? One potential explanation is that children have an underlying bias that leads children to ask the types of questions they do and categorize the way they do. This bias may be age-related in nature. For example, the younger children in this study may be more biased to focus on surface-level, feature-based information. This may lead them to ask questions about feature-related information. Indeed, research has found that children’s categorization behavior is initially based on perceptual similarity, then gradually changes across development to rely on more abstract category-based similarities as children get older (Badger and Shapiro, 2012; Sloutsky et al., 2015). This developmental change in the type of information children prioritize might also apply to their inquiry behavior: in both experiments, we found age-related differences in children’s question-asking. We found a positive correlation between children’s age and the number of category-based questions asked and a negative correlation between age and the number of feature-based questions asked. That is, children were more likely to ask category-based questions and less likely to ask feature-based as they got older, suggesting that there are developmental changes in the questions that children ask.

A second potential explanation for the results in Experiment 2 is that when children’s questions do not receive answers, they may be answering them mentally. That is, an underlying bias toward certain types of information may bias how children answer their own questions, and these internal answers may then impact their categorization behavior. For example, if a child is particularly interested in how and why objects look the way they do, this may lead them to ask a question such as “Do all wugs have orange feet?.” Then, they may mentally answer their question by deciding that all wugs must have feet that are the same shape and color. Thus, when they see a wug with different feet and are asked “Is this a wug or is this something else?,” they will use this internal answer to categorize all wugs with different feet as part of a different category. If this is true, then children’s question asking may not have a direct impact on their categorization; instead, children’s internally generated answers to their questions may be an intermediate step.

As this work was a first step toward studying the act of question asking in the absence of answers, this study also reveals that there is still plenty we do not know about why children ask the questions they do. Specifically, this work was correlational in nature; we do not yet know whether a causal relation exists between children’s questions and their categorization behavior or the direction of this causal relation. Do children’s question themselves truly guide their thinking and behavior? Or do these questions instead reflect a general sense of how children categorize their world? That is, we do not know whether children’s questions change categorization behavior, or whether children’s categorization behavior changes their questions, whether this relation is bidirectional, or whether this relation is only correlational and not causal. Thus, a new direction for future research is to identify the single or multi-step mechanism(s) that explain relations between question asking and categorization. We predict that this relation is bidirectional and multi-step in nature. That is, children’s questions may impact their categorization behavior due to an underlying bias, and their categorization behavior reinforces what information they choose to focus on in subsequent trials.

A final possibility is that the impact of children’s questions may vary based on the context in which they are asking the questions. For instance, there is evidence from previous work (e.g., Alvarez and Booth, 2016; Booth et al., 2020) that there are individual differences in children’s interest in certain types of information, such as causal information, which may impact which types of questions they ask (i.e., causal questions). Moreover, when Chouinard (2007) reported that children only receive answers to questions 71% of the time, this was based only on younger children and on data collected in a naturalistic setting. However, the proportion of questions that do not receive answers may vary based on age or setting, such as a formal classroom setting. Children may ask different types of questions, remember a different amount of questions, or categorize differently depending on how many questions are left unanswered. Thus, future research should examine settings outside of the lab setting in the current study, where all questions were unanswered, to determine how the question asking, memory for questions, and categorization may differ based on these contexts.

On a related note, the social context in which children asked questions may have also influenced their inquiry behavior. First, children in both experiments were prompted by an experimenter to ask questions. We may have seen different results in a motivated-questioning paradigm, where children had the freedom to generate questions spontaneously, rather than in our forced-questioning paradigm. For example, children in a motivated-questioning paradigm may have chosen to ask different types of questions they were more interested in gaining information from other than the feature or category-based questions we observed. Second, the social dynamics of this forced-questioning paradigm may have influenced children’s question-asking. In both experiments, the experimenter made children ask questions and proceeded to not answer them. This dynamic is likely not the same context in which children’s questions typically remain unanswered; children often ask questions to parents or teachers who are facing distractions and thus cannot address each question. In this context, children may have perceived the experimenter as a poor conversational partner and wondered why the experimenter did not answer their questions despite appearing engaged with them. Therefore, children may have assumed that the experimenter was a poor informant and thus chose not to challenge them by asking simpler questions such as feature-based questions, rather than exerting more cognitive effort to ask diverse types of questions. Indeed, children adjust their inquiry behavior based on their perceptions of informants’ knowledge (Mills et al., 2010, 2011; Mills and Landrum, 2016). As context is a limitation of the current research, an important next step in this work is to examine the impact of question asking in a more diverse set of contexts.

A challenge for the field moving forward is to come up with ways to compare cognitive outcomes between questions with and without an answer. In this work, we chose not to include an answered questions condition because there are major theoretical and methodological concerns in doing so; namely, it is impossible to do so in an unconfounded way. For instance, it would be impossible to ensure that all children receive the exact same answers, as the questions asked will differ among children. As previous research suggests, these answers will likely guide children’s questions differently, making it more difficult to isolate the potential impact of questions themselves. Moreover, in a condition where children receive answers, we cannot determine whether the answer alone, the question alone, or an interaction between the question and answer contributed to children’s cognition. Researchers should not directly compare unanswered questions to answered questions until we more clearly understand interactions between the questions that are asked and the answers themselves.

In sum, this study expands our knowledge of the role of children’s questions in cognitive development by demonstrating that children’s question asking relates to their working memory and categorization. While we know that children ask questions for information gain, this study shows that question-asking itself, in the absence of information gain, relates to children’s thinking. Indeed, future research should examine questions without answers to determine the mechanisms underlying question-asking, and whether these processes are causal in nature. This approach will allow the field to build a theory that precisely delineates the cognitive changes as a result of asking a question and the cognitive changes due to gaining information.

## Data availability statement

The raw data supporting the conclusions of this article will be made available by the authors, without undue reservation.

## Ethics statement

The studies involving human participants were reviewed and approved by the University of Wisconsin-Madison Education and Social/Behavioral Sciences Institutional Review Board. Written informed consent to participate in this study was provided by the participants’ legal guardian/next of kin.

## Author contributions

EL and HV: study conception and design, analysis and interpretation of results, and draft manuscript preparation. EL: data collection. All authors reviewed the results, contributed to the article, and approved the submitted version.

## Funding

This work was supported by the Wisconsin Alumni Research Foundation, the Wisconsin Center for Education Research, and the National Science Foundation under grant #1561531.

## Conflict of interest

The authors declare that the research was conducted in the absence of any commercial or financial relationships that could be construed as a potential conflict of interest.

## Publisher’s note

All claims expressed in this article are solely those of the authors and do not necessarily represent those of their affiliated organizations, or those of the publisher, the editors and the reviewers. Any product that may be evaluated in this article, or claim that may be made by its manufacturer, is not guaranteed or endorsed by the publisher.

## Footnotes

^1^These children did not appear to be qualitatively different than children who followed directions. For instance, the 10 children that did not ask questions remembered the majority of their observation statements (*M* = 78.6%, *SD* = 4.79, range = 20–35), suggesting similar memory abilities to children who did ask questions during the experiment.
