# Supplementary material for: “What makes this a wug?” Relations among children’s question asking, memory, and categorization of objects
Source: Front Psychol. 2022 Aug 11;13:892298. doi: 10.3389/fpsyg.2022.892298 (PMC9403714; doi:10.3389/fpsyg.2022.892298)
Supplement: Supplementary file 1 [file Data_Sheet_1.docx]

**Appendix A**

*Demographic information for Experiments 1 and 2*

|  | *Exp. 1 (N=39)* | *Exp. 2*  *(N=39)* | | |  |  |  | |  |  |
| --- | --- | --- | --- | --- | --- | --- | --- | --- | --- | --- |
| *Race*  American Indian/Alaska Native  Asian  Black/African American  Native Hawaiian/Other Pacific  Islander  White  More than one race  Prefer not to disclose  *Ethnicity*  Hispanic or Latino  Not Hispanic or Latino  Prefer not to disclose  *Household Income*  Less than $24,999  $25,000 to $49,999  $50,000 to $99,999  $100,000 or more  Prefer not to disclose  *Parental Education*  Some high school  High school graduate  Trade/Technical/Vocational  Some college  College graduate  Postgraduate  Prefer not to disclose  *Language History*  English  Spanish  Mandarin  Other | *Number*  1  0  1  0  31  5  0  2  35  1  2  1  4  28  4  0  1  1  1  4  32  0  39  1  0  0 | *%*  2.56  0  2.56  0  79.49  12.82  0  5.13  89.74  2.56  5.13  2.56  10.26  71.79  10.26  0  2.56  2.56  2.56  10.2  82.05  0  100  2.56  0  0 | *Number*  0  2  2  0  29  6  0  5  34  0  2  2  9  20  6  1  1  3  1  9  24  0  39  4  0  1 | *%*  0  5.13  5.13  0  74.36  15.38  0  12.82  87.18  0  5.13  5.13  23.08  51.28  15.38  2.56  2.56  7.69  2.56  23.08  61.54  0  100  10.26  0  2.56 | | | |  | |  |

**Appendix B**

*Examples of question types coded for in Experiments 1 and 2*

| *Question Type* | *Example* |
| --- | --- |
| Feature  Category  Function  Behavior  Ambiguous  Location  Creation  Social Relevance | “What is that green thing on the top?”  “Is it a type of bird?”  “What is it used for?”  “Does it talk?”  “What is it?”  “Where does it live?”  “How was it made?”  “Do children like it?” |

**Appendix C**

*Numbers of Children Exhibiting Different Patterns of Stability in Question-Asking (Experiment 1)*

| *Stability of Question-Asking* | *N* | *%* |
| --- | --- | --- |
| No change in feature or category-based questions | 9 | 21.43% |
| Asked more feature-based questions over time | 18 | 42.86% |
| Asked fewer feature-based questions over time  No change in feature-based questions only  Asked more category-based questions over time | 9  15  14 | 21.43%  35.71%  33.33% |
| Asked fewer category-based questions over time  No change in category-based questions only | 12  16 | 28.57%  38.10% |

**Appendix D**

*Mean Numbers of Question Types Asked in Increasing or Decreasing Quantity Over Time (Experiment 1)*

| *Stability of Question-Asking* | *M* | *SD* |
| --- | --- | --- |
| Asked more feature-based questions over time | 2.65 | 2.74 |
| Asked fewer feature-based questions over time  Asked more category-based questions over time | 1.67  2.71 | 0.70  1.68 |
| Asked fewer category-based questions over time | 2.18 | 1.40 |

**Appendix E**

*Order Effects in Children’s Working Memory for Questions*

| *Question Number* | *Mean # Remembered* | *SD* | *t* | *CI lower* | *CI upper* | *p* | *d* |
| --- | --- | --- | --- | --- | --- | --- | --- |
| 1 | 9.02 | 2.43 | -2.09 | 0.01 | 0.64 | .043 | 0.33 |
| 2  3 | 9.02  9.83 | 2.61  2.52 | -3.19 | 0.17 | 0.82 | .003 | 0.50 |

*Note. In order of appearance, t-tests are for memory for the third versus first question and memory for the third versus second question.*

**Appendix F**

*Numbers of Children Exhibiting Different Patterns of Stability in Question-Asking (Experiment 2)*

| *Stability of Question-Asking* | *N* | *%* |
| --- | --- | --- |
| No change in feature or category-based questions | 12 | 29.27% |
| Asked more feature-based questions over time | 18 | 43.90% |
| Asked fewer feature-based questions over time  No change in feature-based questions only  Asked more category-based questions over time | 8  15  10 | 19.51%  36.59%  24.39% |
| Asked fewer category-based questions over time  No change in category-based questions only | 9  22 | 21.95%  53.66% |

**Appendix G**

*Mean Numbers of Question Types Asked in Increasing or Decreasing Quantity Over Time (Experiment 2)*

| *Stability of Question-Asking* | *M* | *SD* |
| --- | --- | --- |
| Asked more feature-based questions over time | 3.06 | 3.13 |
| Asked fewer feature-based questions over time  Asked more category-based questions over time | 1.88  1.70 | 1.36  1.06 |
| Asked fewer category-based questions over time | 2.22 | 1.09 |

**Appendix H**

*Number of Endorsements for Objects in the Categorization Task*

| *Number of feature changes from target item* | *Mean # of Endorsements* | *SD* | *N (children that endorsed)* | *% (children that endorsed)* |
| --- | --- | --- | --- | --- |
| Did not endorse any items  1 | -  7.67 | -  4.50 | 11  6 | 26.8%  14.6% |
| 2  3 | 4.28  3.83 | 4.33  4.42 | 4  4 | 9.8%  9.8% |
| 4 | 2.78 | 3.87 | 16 | 39.0% |

**Appendix I**

*Pairwise Comparisons for Objects in the Categorization Task*

| *Number of feature changes from target item* | *Mean Difference* | *SE* | *p* | *CI lower* | *CI upper* |
| --- | --- | --- | --- | --- | --- |
| 1 vs 2 | 4.04 | .334 | < .001 | 3.001 | 5.079 |
| 1 vs 3  1 vs 4 | 4.56  5.76 | .397  .504 | < .001  < .001 | 3.327  4.194 | 5.793  7.326 |
| 2 vs 3  2 vs 4  3 vs 4 | 0.52  1.72  1.20 | .247  .356  .143 | .331  .002  < .001 | -.247  .613  .757 | 1.287  2.827  1.643 |

**Appendix J**

*Mixed-Effects Logistic Regression Predicting Likelihood of Category Endorsement*

|  | *b* | *CI lower* | *CI upper* | *z* | *p* |
| --- | --- | --- | --- | --- | --- |
| MODEL 1: Age included |  |  |  |  |  |
| Age (months) | .062 | -.011 | .134 | 1.667 | .095 |
| Number of feature questions asked | -.034 | -.044 | -.025 | -7.039 | < .001 |
| Number of category questions asked | .116 | .091 | .141 | 9.070 | < .001 |
| Number of feature changes from  target item | -.573 | -.669 | -.478 | -11.764 | < .001 |
|  |  |  |  |  |  |
| MODEL 2: Age excluded |  |  |  |  |  |
| Number of feature questions asked | -.036 | -.046 | -.027 | -7.678 | < .001 |
| Number of category questions asked | .119 | .094 | .144 | 9.430 | < .001 |
| Number of feature changes from  target item | -.572 | -.669 | -.478 | -11.759 | < .001 |
